# Immune-Related Functions of Heme Oxygenase-1

**DOI:** 10.3390/antiox12071322

**Published:** 2023-06-22

**Authors:** Elias A. Lianos, Maria G. Detsika

**Affiliations:** 1 Veterans Affairs Medical Center and Virginia Tech, Carilion School of Medicine, Salem, VA 24153, USA; elias.lianos@va.gov; 2 1st Department of Critical Care Medicine & Pulmonary Services, GP Livanos and M Simou Laboratories, Evangelismos Hospital, National and Kapodistrian University of Athens, 10675 Athens, Greece

Heme oxygenase (HO)-1 is a well-known cytoprotective enzyme due to its enzymatic action, which involves the catalysis of heme into anti-apoptotic and antioxidant molecules such as bilirubin, biliverdin and CO. Apart from heme, its natural inducer and substrate, HO-1 responds to numerous stimuli and is, therefore, implicated in multiple cellular, molecular and immunological pathways. Its role as a potential immunoregulator has been studied both via its by-products and via the direct involvement of HO-1 in immune-related cellular functions and responses. The *Antioxidants* journal Special Issue titled “Heme Oxygenase (HO)-1 as an Immunoregulator in Health and Disease” aims to highlight its role in immune-related pathways. This Special Issue includes twelve articles, eight of which are research articles reporting recent advances in research on the role of HO-1 in various immune-related mechanisms, and four of which are review articles that provide detailed, up-to-date information and evidence on its role as an immunoregulator in different diseases or health systems.

Cannedo-Marroquin et al. described the increase in the lung HO-1 expression of mice primarily infected with human respiratory syncytial virus (hRSV) and subsequently with a *Mycobacterium bovis* (*M. bovis*) strain referred to as *Bacillus Calmette-Guerin* (BCG) [1]. In a series of elegantly performed experiments, the study showed that a pre-infection with hRSV promoted lung pathology such as increased infiltration of innate immune cells, including interstitial and alveolar macrophages, during a subsequent mycobacterial challenge, thus impairing pulmonary immune responses and promoting secondary mycobacterial colonization, which was also associated with increased lung tissue HO-1 expression.

The involvement of HO-1 in viral infection was also explored in the study by Detsika M. G. and colleagues, who investigated changes in the expression of HO-1 in COVID-19 [2]. The study assessed HO-1 mRNA levels in COVID-19 patients with severe and critical illness and found that HO-1 levels increased in critically ill patients. This increase was accompanied by an increase in HO-1 expression at the tissue level in critically ill COVID-19 patients and was associated with poor prognosis.

The role of HO-1 in COVID-19 infection was further analyzed by Toro et al. [3]. Their review article consists of a thorough description of the anti-inflammatory and antiviral properties of HO-1 and its consequent potential for combating clinical manifestations of the disease. 

Fitzgerald H.K. et al. explored the potential of *Trypanosoma brucei* (*T. brucei*)-derived ketoacids indole pyruvate (IP) and hydroxyphenylpyruvate (HPP) to induce HO-1 and modulate CD4^+^ T cell responses [4]. The study reported upregulation of HO-1 by IP and HPP through Nrf2 in human dendritic cells (DC) associated with decreased DC maturation and pro-inflammatory cytokine production, thus affecting CD4^+^ T cell differentiation. 

The role of HO-1 as an immunomodulator was also assessed in a model of parasitic disease [5]. Costa et al. showed that mice infected with *Fasciola hepatica* (*F. hepatica*), a fluke that infects livestock and humans, causing fasciolosis, exhibited increased HO-1 expression in peritoneal antigen-presenting cells (APCs). The later produced decreased levels of reactive oxygen and nitrogen species, and their presence was associated with increased levels of regulatory T cells in an IL-10 activity-dependent manner.

The effect of the HO-1 pathway on inflammation was also assessed by Hirvonen et al., whose work involved healthy volunteers and revealed that the administration of D-glyceric acid activated mitochondrial metabolism and reduced inflammation [6]. Measurement of the HO-1 reaction by-products showed increased blood bilirubin levels, which were associated with ameliorated inflammation and lower levels of blood triglycerides.

Another study utilized CO-releasing molecules (CORMs) on primary tenocytes in order to investigate their potential to reduce inflammation [7]. Appetecchia et al. treated primary tenocytes with various concentrations of specific CORMs previously tested for their efficacy to produce efficient amounts of CO, an important HO-1 reaction by-product. The results of the study showed an improvement in tendon homeostasis and a reduction in PGE2 secretion.

The impact of HO-1 and its reaction by-products, especially bilirubin, is described in detail in the review article by Thomas D. T. and colleagues [8]. The article depicts the function of bilirubin as an antioxidant and metabolic hormone and how the HO-1–BVRA–bilirubin–PPAR axis influences inflammation and metabolic function and interacts with exercise to improve the outcomes of weight management.

An additional role of HO-1 as a potential modulator of the expression of proteins regulating the activation of the complement cascade was investigated by Detsika et al. [9]. Using tissue samples obtained from transgenic rats with complete HO-1 deficiency, it was shown that la ack of HO-1 reduced decay accelerating factor (DAF) expression in kidney, liver and lung tissue and CR1-related gene/protein Y (Crry) expression in kidney and liver tissue. The reduction in kidney DAF and Crry expression resulted in an increase in C3b deposition in kidney tissue, thus indicating an additional immunomodulatory role of HO-1 as a complement cascade regulator. 

The important relationship of HO-1 and kidney disease via its potential as an immunomodulator is described in detail in the review article by Athanassiadou and colleagues [10]. The article serves as an up-to-date overview on the involvement of HO-1 in kidney disease through its effect on immune pathways and responses. 

Similarly, in the review article by Xia and Zhong, the immunomodulatory role of HO-1 in allergic airway inflammation is described in detail [11]. Specifically, the anti-inflammatory effect of HO-1 in different stages of airway inflammation via the regulation of various immune cells, including dendritic cells, mast cells, basophils, T cells and macrophages, is discussed. 

Finally, the anti-tumoral role of HO-1 via different mechanisms, including immune mediated pathways, was demonstrated in the study by Lage-Vickers et al. [12]. The study employed a proteomics approach and analyzed prostate cancer proteomic data in order to assess the significance of HO-1 interactors. 

In conclusion, this Special Issue emphasizes the potential of HO-1 to modulate various immune cellular and physiological pathways, thus strengthening the evidence for its promising role as a putative target leading to innovative avenues of therapeutic strategies. 
